# Quality of life among patients undergoing bariatric surgery: associations with mental health- A 1 year follow-up study of bariatric surgery patients

**DOI:** 10.1186/1477-7525-9-79

**Published:** 2011-09-26

**Authors:** Haldis Ø Lier, Eva Biringer, Oddbjørn Hove, Bjarte Stubhaug, Tone Tangen

**Affiliations:** 1 Section of Mental Health Research, Haugesund Hospital, Helse Fonna HF. P.O.Box 2170, N-5504 Haugesund, Norway; 2 Section of Psychiatry Institute of Clinical Medicine University of Bergen, Bergen, Norway

## Abstract

**Background:**

Preoperative mental health seems to have useful predictive value for Health Related Quality of Life (HRQOL) after bariatric surgery. The aim of the present study was to assess pre- and postoperative psychiatric disorders and their associations with pre- and postoperative HRQOL.

**Method:**

Data were assessed before (n = 127) and one year after surgery (n = 87). Psychiatric disorders were assessed by Mini International Neuropsychiatric Interview (M.I.N.I.) and Structured Clinical Interview (SCID-II). HRQOL was assessed by the Short Form 36 (SF-36) questionnaire.

**Results:**

Significant improvements were found in HRQOL from preoperative assessment to follow-up one year after surgery. For the total study population, the degree of improvement was statistically significant (*p *values < .001) for seven of the eight SF-36 subscales from preoperative assessment to follow-up one year after surgery. Patients without psychiatric disorders had no impairments in postoperative HRQOL, and patients with psychiatric disorders that resolved after surgery had small impairments on two of the eight SF-36 subscales compared to the population norm (all effect sizes < .5) at follow-up one year after surgery. Patients with psychiatric disorders that persisted after surgery had impaired HRQOL at follow-up one year after surgery compared to the population norm, with effect sizes for the differences from moderate to large (all effect sizes ≥ .6).

**Conclusion:**

This study reports the novel finding that patients without postoperative psychiatric disorders achieved a HRQOL comparable to the general population one year after bariatric surgery; while patients with postoperative psychiatric disorders did not reach the HRQOL level of the general population. Our results support monitoring patients with psychiatric disorders persisting after surgery for suboptimal improvements in quality of life after bariatric surgery.

**Trial Registration:**

The trial is registered at http://www.clinicaltrials.gov prior to patient inclusion (ProtocolID16280).

## Introduction

Bariatric surgery is currently considered the most effective treatment to achieve sustained weight loss in patients with severe obesity [1]. Candidates for surgery are patients with BMI > 40 kg/m^2 ^or BMI > 35 kg/m^2 ^with serious somatic obesity-related disorders [1,2]. Several studies have shown improvement in HRQOL after bariatric surgery [3-5]. Improvement in HRQOL is reported shortly after surgery [6], and HRQOL is comparable to the general population two years after surgery [4]. However, variability exists in the postoperative outcomes [2,7]. In a study by Karlsson et al, about two-thirds of the surgically treated patients reported maintained weight loss of more than 10%, and this seemed to be sufficient for positive effects on quality of life ten years after surgery [3]. The reasons for variability in bariatric surgery outcomes appear to be related in part to inadequate behavioural and emotional changes [2,8].

Patients with obesity have increased prevalence of psychiatric disorders compared to the general population [9-11]. In obese patients waiting for bariatric surgery, prevalence rates of life time psychiatric disorders vary from 37% to 73% and prevalence rates of current psychiatric disorders vary from 20% to 56% [9,10,12,13]. Comorbid psychiatric disorders generally aggravate symptoms and adversely affect treatment outcome in various somatic disorders like diabetes, pulmonary diseases and heart diseases [14-16]. Further, personality appears to have substantial influence on health behaviour [17,18]. Accordingly, several studies have focused on the influence of comorbid psychiatric disorders on the results of bariatric surgery [19-22]. Two prospective studies, that included structured interviews in assessing psychiatric disorders, examined the role of psychiatric comorbidity for treatment outcome in bariatric surgery [19,20]. One study found that patients with a life time history of depression or anxiety had less weight loss six months after surgery compared to patients who had never had such disorders [19]. The other study found that having more than two preoperative psychiatric disorders compared to having none or only one preoperative psychiatric disorders was associated with less weight loss after surgery [20]. Kinzl et al. also demonstrated that a combination of Axis I and Axis II disorders predicted poorer HRQOL after surgery [23]. A number of studies have examined the role of preoperative psychological factors in predicting outcome of bariatric surgery [22,24-26]. The association between preoperative mental health and HRQOL, mental wellbeing and physical wellbeing after bariatric surgery is found in earlier research [22,27,28], while no consistent association between preoperative comorbid psychiatric disorders and postsurgical weight loss has been reported [27].

Psychological symptoms that persist or reoccur after bariatric surgery might have stronger impact on the course of weight loss and quality of life than preoperative psychiatric disorders [21,29,30]. One study by de Zwaan et al. found that postoperative Axis I psychiatric disorders were related to weight loss [30]. White et al. reported that postoperative, but not preoperative loss of control over eating was a negative predictor for postsurgical weight loss and psychosocial outcomes at follow-up one and two years after surgery [29]. Further, improvements in HRQOL one year after surgery were found to be largely related to improvement of depressive symptoms [21].

Standardized clinical interviews are generally considered as the most reliable and valid method for the assessment of psychiatric disorders [31,32]. To our knowledge, only one published study has assessed comorbid psychiatric disorders by standardized clinical interviews both prior to and after surgery [30], and no published studies have assessed the association between postoperative Axis I and Axis II psychiatric disorders assessed by structured clinical interviews and quality of life.

In the present study pre- and postoperative comorbid psychiatric disorders and their associations with HRQOL were examined. We hypothesised that patients with comorbid preoperative psychiatric disorders that persist after surgery would have more impaired HRQOL compared to patients without psychiatric disorders and patients with preoperative psychiatric disorders that resolve after surgery.

## Method

### Study population

A total of 169 patients with severe obesity were evaluated for the study. They were all referred from general practitioners (GPs) to the Department of Surgery at Haugesund Hospital on the West coast of Norway. This region includes both urban and rural areas. The bariatric procedure at Haugesund hospital is laparoscopic Roux-en-Y gastric bypass. Nineteen patients were excluded (did not meet for psychiatric assessment (n = 3), psychiatric assessment not relevant (n = 16: pregnant (n = 3), bariatric surgery at private hospitals (n = 7), did not want bariatric surgery (n = 6)). Two patients refused to participate in the study, three patients retracted their consent, and four patients did not fulfil inclusion criteria. Mean age was significantly higher in the patients who refused to participate compared to the study population (56.4 vs. 42.0 years, *p *< .001), there was no difference in female/male ratio or in BMI. Exclusion criteria were severe psychopathology (psychotic disorder, severe mood disorder), severe eating disorder, risk of suicidal behaviour, severe substance abuse, or severe cognitive dysfunction, all based on clinical judgment. Four patients, three women and one man (with mean age 51.0 years), were excluded from this study due to severe mood disorder and severe eating disorder. The patients were receiving inadequate clinical treatment, and we recommend more adequate treatment for these disorders before surgery. One-hundred-and-forty-one patients (84%) were included in the study. One-hundred-and-twenty-seven patients (90%) received surgical treatment. We were able to get some information from 91 patients (72%) at follow-up one year after surgery. However, four of these patients did not complete the psychiatric assessment (not possible to make an appointment for the diagnostic interviews), hence, postoperative data on both psychiatric disorders and quality of life were available from 87 patients (69%).

### Assessment

All patients were assessed by face to face interviews by a psychiatrist (1^st ^author) at preoperative assessment. At follow-up one year after surgery, assessment was done by face-to-face interviews or telephone interviews. Patients were interviewed by M.I.N.I. and SCID-II and filled in self-report instruments measuring HRQOL at both preoperative assessment and at follow-up one year after surgery. Body weight was measured to the nearest 0.5 kg using an electronic scale or self-reported (telephone interviews). Body height was self-reported.

### Instruments

#### Psychiatric evaluation

The Mini International Neuropsychiatric Interview (M.I.N.I.) for DSM-IV (Norwegian version 5.0.0) was used to establish current psychiatric diagnosis and psychiatric comorbidity for Axis I symptom disorders [33]. The M.I.N.I has well-established reliability and validity [34,35]. Personality diagnoses were obtained using a shortened Norwegian version of the Structured Clinical Interview for DSM-IV Axis II [36,37].

### Health related quality of life

Short Form Health Survey 36 (SF-36) is a well validated self-rating questionnaire that measures HRQOL [38]. The SF-36 assesses eight dimensions of health, each ranging from 0-100; higher scores represent better health. The SF-36 comprises 36 items that describe the following eight dimensions of functioning: Physical Functioning, Physical Role Limitation, Bodily Pain, General Health, Vitality, Social Functioning, Emotional Role Limitation, and Mental Health. The validity of the Norwegian version of SF-36 has been tested and found satisfactory, and norm data were obtained from this Norwegian study [39]. At baseline six of the patients without psychiatric disorders, two of the patients with psychiatric disorders that resolve after surgery, one of the patients with psychiatric disorders that persist after surgery had one or more missing responses on one or more items of the questionnaire. At one year follow-up missing responses for these three groups were six, one, and four, respectively. More than 50% missing items in a subscale caused the whole subscale to be missing. At baseline two of the patients without psychiatric disorders, two of the patients with psychiatric disorders that resolve after surgery, and none of the patients with psychiatric disorders that persist after surgery had one or more missing subscales. At one year follow-up these three groups had two, one and no missing subscales, respectively.

### Statistical methods

Group comparisons between groups with and without comorbid psychiatric disorders were performed using Pearson's Chi-square Test and Mann-Whitney U Test (continuous variables were not normally distributed). Wilcoxon Signed Rank Test was used to analyze differences in SF-36 subscales from preoperative assessment to assessment one year after surgery. McNemars test for dependent proportions was used to examine if preoperative and postoperative prevalence of psychiatric disorders were significantly different from each other.

The Kruskal-Wallis Test was conducted to evaluate differences among three groups of the study population (patients without psychiatric disorders, patients with psychiatric disorders that persist after surgery, and patients with psychiatric disorders that resolve after surgery) with regard to pre- and postoperative HRQOL and the change in score for HRQOL (preoperative score minus postoperative score of SF-36 subscales).

HRQOL in groups with and without comorbid psychiatric disorders was compared to the population norm. The population norm data were adjusted by age and gender [40]. We calculated effect sizes to compare the mean values of SF-36 subscales in this study population to the population norm by subtracting the mean scores of the population norm from the mean score of the patient group divided by the standard deviation of the patient group. Effect sizes < .2 are considered trivial, from. 2 to < .5 small, from .5 to < .8 moderate, and > .8 large [41].

To examine if change in SF-36 subscales from preoperative assessment to follow-up one year after surgery reflected a regression-to-mean phenomenon, we used the method described by Altman [42]; calculated the average of the initial and the final measurement and then calculated the correlation between this quantity and the observed change. Effect size indices (*d *) were computed for the difference in mean scores of SF-36 subscales from preoperative assessment to follow-up for all three groups. Effect sizes < .2 are considered trivial, from .2 to < .5 small, from .5 to < .8 moderate, and > .8 large [41,43]. Further, Z-scores for change for each SF-36 subscales within the three patient groups are presented.

A one way analysis of variance was conducted to evaluate differences in change in BMI from preoperative assessment to follow-up within the three groups. Further, correlation coefficients (Spearman correlation coefficients) were computed among the change scores of SF-36 subscales and change in BMI.

Tests were two-tailed as correlations could be bidirectional. A Bonferroni adjustment for multiple testing was done, setting the α-level to .01.

SPSS 17.0 for Windows was used for the statistical analyses.

### Ethical issues

All participants provided written informed consent prior to assessment. The study was approved by the Regional Committees for Medical and Health Research Ethics and the Norwegian Social Science Data Services (NSD). The trial was registered at http://www.clinicaltrials.gov prior to patient inclusion (ProtocolID 16280). The study was performed in accordance with The Helsinki Declaration of the World Medical Association Assembly.

The study was supported by a grant from the Western Regional Health Authority, Norway.

## Results

### Demographic characteristics and clinical data

Demographic and clinical characteristics are reported in Table 1 and 2. There were 94 (74%) female patients, mean age was 41 years (SD = 10.3), and mean BMI 45.3 kg/m^2 ^(SD = 5.2). Patients with preoperative comorbid psychiatric disorders had significantly lower level of education and were significantly less often employed compared to patients without preoperative comorbid psychiatric disorders (Table 1).

**Table 1:** Sociodemographic information of 127 patients waiting for bariatric surgery

	Total sample (N = 127)			Patients with comorbid psychiatric disorders (N = 61)			Patients without comorbid psychiatric disorders (N = 66)			Group comparison*
	**Mean**	**SD**	**Range**	**Mean**	**SD**	**Range**	**Mean**	**SD**	**Range**	***p***
	
Age (years)	41.3	10.3	22-65	41.0	11.3	22-65	41.7	9.3	23-61	.704
Preoperative BMI, kg/m^2^	45.3	5.2	34.5-64.6	44.9	5.7	36.5-64.7	45.6	4.7	34.5-59.2	.277
Postoperative BMI, kg/m^2^	31.6	4.6	21.2-44.4	30.1	4.9	21.2-44.4	32.1	4.1	23.5-41.7	.023
	N (%)			N (%)			N (%)			
	
Gender, female	94 (74)			47 (77)			47 (71)			.454
Education (N = 124)										
< 10 years	42 (33)			28 (46)			14 (21)			.003
10-13 years	60 (47)			27 (44)			33 (50)			.518
> 13 years	23 (18)			4 (7)			19 (29)			.001
Work status (N = 122)										
Employed	71 (60)			25 (45)			47 (74)			.001
Civil status (married/cohabitant)	80 (65)			33 (55)			47 (74)			.032

*Mann-Whitney U Test and Pearson's Chi-square Test

### Comorbid psychiatric disorders

Sixty-one patients (48%) fulfilled the diagnostic criteria for at least one current Axis I or Axis II disorder (DSM-IV) preoperatively and 18% fulfilled the diagnostic criteria for at least one Axis I or Axis II disorder one year after surgery. The prevalence of depressive disorders, anxiety disorders, and personality disorders were 22%, 30%, and 24% respectively at preoperative assessment and 11%, 13% and 8% respectively at postoperative assessment (*p- *values for the differences < .05). (For prevalence of specific preoperative psychiatric disorders see Lier et al. (2011) [44]). Forty-two percent of the patients with a preoperative psychiatric disorder still had a psychiatric disorder at follow-up one year after surgery (*p <*.001, McNemars test for dependent proportions).

### Pre- and postoperative HRQOL

At preoperative assessment effect sizes for the differences in score on SF-36 between the study population and norm data for the general population were large (> .8) for five of the eight subscales of SF-36 (Physical Functioning, Physical Role Limitation, Bodily Pain, General Health, and Vitality), moderate (from .3 to .6) for two subscales (Social Functioning and Mental Health), and trivial for one (Emotional Role Limitation). One year after surgery, effect sizes for the difference in score on SF-36 between this study population and population norm were trivial (< .2) for seven of the eight subscales (Physical Functioning, Physical Role Limitation, General Health, Vitality, Social Functioning, Mental Health, and Emotional Role Limitation) and moderate ( .3) for one subscale (Bodily Pain).

For the study population there was a significant improvement in score on all SF-36 subscales (*p *< .001), except for the Emotional Role Limitation subscale (*p *= .201) from preoperative assessment to follow-up one year after surgery (Table 2).

**Table 2:** Pre- and postoperative HRQOL in the study population

	Preoperative Mean (SD) N = 127	Postoperative Mean (SD) N = 87	Difference in scores *	Population norm **
**SF-36 Physical Functioning Subscale** Effect size***	56.8 (23.3) -1.41	87.5 (19.1) - .11	< .001	89.6
**SF-36 Physical Role Limitation Subscale** Effect size***	40.3 (39.1) -1.06	74.7 (39.3) - .18	< .001	81.9
**SF-36 Bodily Pain Subscale** Effect size***	46.3 (25.1) -1.18	66.5 (29.9) - .31	< .001	75.8
**SF-36 General Health Subscale** Effect size***	47.8 (22.2) -1.38	78.7 (23.0) .01	< .001	78.5
**SF-36 Vitality Subscale** Effect size***	40.9 (19.4) - .95	61.1 (21.4) .09	< .001	59.3
**SF-36 Social Function Subscale** Effect size***	71.5 (25.0) - .57	89.0 (19.9) .16	< .001	85.9
**SF-36 Emotional Role Limitation Subscale** Effect size***	78.8 (36.5) - .12	83.7 (34.4) - .15	.201	83.4
**SF-36 Mental Health Subscale** Effect size***	73.3 (16.1) - .32	80.9 (17.2) .16	< .001	78.4

*Difference in scores from preoperative assessment to postoperative assessment, Wilcoxon Signed Rank Test

** Population norm [39], adjusted for age and gender.

*** Effect size is calculated by subtracting the mean score of the population norm (adjusted for age and gender) from the mean score of the patients group and divided by the standard deviation of the patient group.

### Comorbid psychiatric disorders and pre- and postoperative HRQOL

A Kruskal-Wallis test was conducted to evaluate differences among the three patient groups (patients without psychiatric disorders, patients with psychiatric disorders that persist after surgery, and patients with psychiatric disorders that resolve after surgery) on preoperative HRQOL, postoperative HRQOL and change in HRQOL. There were significant differences in four of the preoperative subscales of SF-36 (Physical Functioning, General health, Social Functioning, Emotional Role Limitation, and Mental Health, all *p *values < .01) and five of the postoperative subscales of SF-36 (Physical Functioning Subscale, General health, Social Functioning, Emotional Role Limitation, and Mental Health, all *p *values < .01) between these three groups. No significant differences were observed between the "change scores" of the subscales of SF-36 between these three groups (all *p *values > .01) (Table 3).

**Table 3:** Pre- and postoperative HRQoL in patients with and without pre- and postoperative comorbid psychiatric disorders

	Patients without psychiatric disorders, N = 49					Patients with psychiatric disorders that resolve after surgery, N = 22					Patients with psychiatric disorders that persist after surgery, N = 16				
	T0* Mean (SD)	T1* Mean (SD)	P**	d ***	**Change (T1-T0) Mean Z-score (SD)**	T0* Mean (SD)	T1* Mean (SD)	P**	d ***	**Change (T1-T0) Mean Z-score (SD)**	T0* Mean (SD)	T1* Mean (SD)	P**	d ***	**Change (T1-T0) Mean Z-score (SD)**

SF-36 Physical Functioning Subscale	60.8 (22.1)	93.0 (12.1)	< .001	1.71	< .01 (1.0)	52.3 (17.0)	82.1 (24.2)	< .001	1.79	.1 (1.0)	46.0 (27.2)	73.6 (25.2)	.001	1.50	- .4 ( .8)
Effect size****	-1.30	.27				-2.2	- .29				-1.6	- .63			
SF-36 Physical Role Limitation Subscale	51.7 (41.0)	83.1 (32.1)	< .001	.78	- .1 ( .9)	35.0 (40.9)	77.4 (41.8)	.006	.91	.3 (1.2)	23.4 (34.7)	54.2 (48.7)	.076	.53	- .1 ( .7)
Effect size****	- .74	.02				-1.13	- .09				-1.68	- .56			
SF-36 Bodily Pain Subscale	51.9 (24.5)	75.1 (26.8)	< .001	.87	.1 (1.0)	37.3 (14.6)	60.5 (31.0)	.01	.76	.2 (1.2)	39.3 (29.9)	50.4 (33.2)	.023	.67	- .2 ( .7)
Effect size****	- .98	- .03				-2.60	- .47				-1.24	- .78			
SF-36 General Health Subscale	56.3 (22.5)	86.8 (13.9)	< .001	1.29	< .01 (1.0)	41.6 (13.5)	76.6 (19.5)	< .001	1.73	.2 ( .8)	34.3 (22.7)	54.4 (34.4)	.033	.75	- .5 ( 1.0)
Effect size****	- .99	.60				-2.67	- .05				-1.94	- .70			
SF-36 Vitality Subscale	43.3 (18.9)	65.5 (19.2)	< .001	.88	< .01 (1.0)	32.5 (17.4)	63.1 (20.2)	< .001	1.40	.5 ( .9)	36.9 (22.9)	45.4 (21.8)	.016	.78	- .3 ( .7)
Effect size****	- .87	.30				-1.60	.14				-1.0	- .66			
SF-36 Social Function Subscale	79.1 (20.2)	95.3 (12.8)	.001	.64	- .1 ( .9)	68.6 (19.7)	94.6 (11.6)	< .001	1.51	.4 ( .7)	49.2 (29.4)	59.4 (26.2)	.053	.51	- .1 (1.2)
Effect size****	- .34	.72				- .89	.72				-1.26	-1.02			
SF-36 Emotional Role Limitation Subscale	87.2 (29.5)	93.0 (20.0)	.103	.25	< .01 ( .7)	78.3 (26.3)	90.5 (30.1)	.170	.38	.3 (1.2)	50.0 (48.7)	47.2 (48.1)	.414	- .22	- .4 ( .9)
Effect size****	.12	.46				- .21	.22				- .68	- .74			
SF-36 Mental Health Subscale	78.7 (12.5)	86.3 (10.5)	< .001	.58	< .01 ( .8)	71.6 (12.8)	85.1 (10.0)	.002	.96	.4 ( .9)	59.5 (21.7)	55.3 (25.5)	.305	- .20	- .4 ( 1.4)
Effect size****	.02	.74				- .57	.62				- .87	- .90			

*T0 = preoperative assessment, T1 = follow-up one year after surgery. **Difference in scores from preoperative assessment to postoperative assessment, Wilcoxon Signed Rank Test. *** Paired-Samples t-test to calculate effect size index d **** Effect size is calculated by subtracting the mean score of the population norm (adjusted for age and gender) from the mean score of the patients group and divided by the standard deviation of the patient group.

To examine if improvement in quality of life reflected a regression- to-mean phenomenon, correlation coefficients were computed between the average of the initial and the final score on each SF-36 subscale and the observed change in each SF-36 subscale. In the group of patients without psychiatric disorders, large correlations were found for three subscales; Physical Functioning (r = .80), General Health (r = .58), and Social Functioning (r = .51). In the group of patients with psychiatric disorders that resolve after surgery, large correlations were found for three subscales; Social Functioning (r = .88), Emotional Role Limitation (r = .64), and Bodily Pain (r = .70). In the group of patients with psychiatric disorders that persist after surgery, no large correlations were found.

Z- scores for change of each of the eight subscales of SF-36 are shown in Table 3. All eight SF-36 subscales showed descriptively greater improvement in the group of patients with psychiatric disorders that resolve after surgery compared to both patients without psychiatric disorders, and patients with psychiatric disorders that persist after surgery, and all eight SF-36 subscales showed descriptively smaller improvement in the group of patients with psychiatric disorders after surgery compared to the patients without postoperative psychiatric disorders.

### Patients without psychiatric disorders and pre- and postoperative HRQOL

Patients without psychiatric disorders had impaired HRQOL on six of the eight subscales (Physical Functioning, Physical Role Limitation, Bodily Pain, General Health, Vitality, and Social Functioning) at preoperative assessment compared to the population norm. The effect sizes for the differences were large (.9 to 1.3) for four subscales (Physical Functioning, Bodily Pain, General Health, and Vitality), moderate (.7) for one subscale (Physical Role Limitation), and trivial to small (< .3) for three subscales (Social Functioning, Emotional Role Limitation, and Mental Health). These patients' scores showed significant improvements on seven of the eight subscales of SF-36 (Physical Functioning, Physical Role Limitation, Bodily Pain, General Health, Vitality, Social Functioning, and Mental Health; all *p *values < .01) from preoperative to postoperative assessment. The effect sizes for the improvement in all SF-36 subscales were moderate to large (*d *> .5) except for the Emotional Role Limitation subscale (*d*= .25). This group of patients had no impairment in HRQOL compared to the population norm at follow-up one year after surgery (Table 3).

### Patients with psychiatric disorders that resolve after surgery and pre- and postoperative HRQOL

Patients with psychiatric disorders that resolve after surgery had impaired HRQOL on all eight subscales at preoperative assessment compared to the population norm. The effect sizes for the differences in HRQOL between this group and the population norm were large (.9- 2.7) for six subscales (Physical Functioning, Physical Role Limitation, Bodily Pain, General Health, Vitality, and Social Functioning), moderate (.6) for one subscale (Mental Health), and small (.2) for one subscale (Emotional Role Limitation). These patients' scores showed significant improvements on seven of the eight subscales of SF-36 (Physical Functioning, Physical Role Limitation, Bodily Pain, General Health, Vitality, Social Functioning, and Mental Health; all *p *values < .01) from preoperative to postoperative assessment. The effect sizes for the improvement in all SF-36 subscales were moderate to large (*d *> .5) except for the Emotional Role Limitation subscale (*d*= .38). They had impaired HRQOL on two subscales (Physical Functioning and Bodily Pain) compared to the population norm (effect sizes .3- .5) one year after surgery (Table 3).

### Patients with psychiatric disorders that persist after surgery and pre- and postoperative HRQOL

Patients with psychiatric disorders that persist after surgery had impaired HRQOL on all eight subscales at preoperative assessment compared to the population norm. The effect sizes for the differences in preoperative HRQOL between this group and the population norm were large (.9- 1.9) for seven subscales (Physical Functioning, Physical Role Limitation, Bodily Pain, General Health, Vitality, Social Functioning, and Mental Health) and moderate (.7) for one (Emotional Role Limitation). The effect sizes for the improvement in all SF-36 subscales were moderate to large (*d *> .5) except for the Mental health subscale and the Emotional Role Limitation subscale (*d*= - .20 and *d *= - .22 respectively). However, in this patient group the improvement from preoperative to postoperative assessment was significant for only one of the eight subscales of SF-36 (Physical Functioning; *p *value < .01), and they had impaired HRQOL on all subscales at follow-up one year after surgery. The effect sizes for the differences in postoperative HRQOL between this group and the population norm were large (.9- 1.9) for two subscales (Social Functioning and Mental Health) and moderate (.6- .7) for six subscales (Physical Functioning, Physical Role Limitation, Bodily Pain, General Health, Vitality, and Emotional Role Limitation) (Table 3).

### Health related quality of life and weight loss in patients with and without comorbid psychiatric disorders

Patients without psychiatric disorders, patients with psychiatric disorders that resolve after surgery, and patients with psychiatric disorders that persist after surgery had preoperative BMI (means) of 46.1, 42.4 and 45.3 respectively, and postoperative BMI of 32.1, 28.6 and 32.9 respectively. There were no significant differences in change of BMI from preoperative assessment to follow-up between the three groups (F(2,78) = 1.2, *p*= .301). Mean score of change of BMI in patients with psychiatric disorders that persist after surgery (Mean = 12.4, SD = 2.7) was not significantly different from patients without psychiatric disorders (Mean = 14.1, SD = 4.0) or patients with psychiatric disorders that resolve after surgery (Mean = 13.8, SD = 3.5). No significant correlation between change of BMI and change in score of the subscales of SF-36 was found in any of the three groups (none of the correlations were significant, all *p *values > .05).

## Discussion

In the present study population there was a significant improvement in HRQOL from preoperative assessment to follow-up one year after bariatric surgery. Patients without postoperative psychiatric disorders had HRQOL comparable to the population norm at follow-up one year after surgery. However, in patients with psychiatric disorders that persist after surgery, only a trend toward improvement could be detected in most of the subscales measuring HRQOL, and these patients still had impaired HRQOL compared to the population norm one year after surgery.

Patients without psychiatric disorders, patients with psychiatric disorders that resolve after surgery, and patients with psychiatric disorders that persist after surgery had impaired quality of life at preoperative assessment. The largest impairments in HRQOL were found in patients with comorbid psychiatric disorders. This association between preoperative comorbid psychiatric disorders and impaired HRQOL is in accordance with earlier research [10,13,21,45]. Kalarchian et al (2007) found that lifetime Axis I disorders were associated with significantly lower scores on each subscale of SF-36 [10]. Mauri et al (2008) found that having both Axis I and II disorders were significantly associated with impaired HRQOL, while having only Axis I or Axis II disorders did not predict lower scores on HRQOL [13].

Forty-two percent of the patients in our sample had a psychiatric disorders that persist after surgery. There was a significant reduction in both Axis 1 and Axis II disorders from preoperative assessment to follow-up. This is in line with previous studies and reviews of studies that have claimed that prevalence rates of Axis I disorders are from 0% to 50% of presurgical status [27]. In a study by de Zwaan et al., prevalence of current depressive disorders and current anxiety disorders were 33% and 17%, respectively, before surgery, and 17%, and 15%, respectively, at follow-up six to twelve months after bariatric surgery [30]. However, in contrast to the finding in our study of a significant reduction in prevalence of personality disorders, earlier research has reported personality pathology as largely unchanged after bariatric surgery [27]. In the present study, avoidant personality disorder was the most prevalent preoperative Axis II disorder [44]. There is significant overlap of diagnostic criteria between social phobia and avoidant personality disorder [46]. And we may speculate that treatment for obesity, which is a stigmatising condition, might reduce social inhibition, negative self- evaluation, and self-criticism and thereby reduce both social phobia and avoidance tendencies. This is in accordance with earlier research: Liebowitz et al. found that effective treatment for social phobia was associated with a reduction in patients who fulfilled the criteria for avoidant personality disorder [47]. However, for some patients (18% in this study), psychopathology seemed not to be related to the degree of obesity and persisted after weight loss.

Patients with postoperative psychiatric disorder had larger impairments in postoperative HRQOL compared to patients without postoperative psychiatric disorders. Few previous studies have assessed this association between post-operative psychological symptoms and outcome of bariatric surgery [21,29,30], and no published studies have presented results on how postoperative psychiatric disorders are related to quality of life after bariatric surgery. However, in a study by Masheb et al., improvement in depressive symptoms from preoperative assessment to follow-up one year after bariatric surgery was found to be a significant predictor for improvement in quality of life [21]. Our results support surveillance of patients with psychiatric disorders that persist after surgery for suboptimal improvements in quality of life after bariatric surgery. Close monitoring of patients after surgery might help to identify patients who could benefit from adjunctive treatment for psychiatric disorders as post-surgery psychiatric symptoms may be a potential mediator for poorer quality of life outcome after surgery.

Improvement in quality of life was not correlated with change in BMI from preoperative assessment to follow-up in any of the three groups (patients without psychiatric disorders, patients with psychiatric disorders that resolve after surgery, and patients with psychiatric disorders that persist after surgery). Further, there was no significant difference in weight loss between these groups. However, patients with psychiatric disorders that persist after surgery had descriptively a smaller change in BMI compared to patients without postoperative psychiatric disorders. Previous studies have reported associations between both weight loss and quality of life [3,48], and between postoperative psychiatric disorders and weight loss [30]. In the study by Karlsson et al., with ten years follow-up, improvements and deteriorations in HRQOL were associated with the magnitude of weight loss or regain [3]. Further, in the study by de Zwan et al., an association between weight loss and depressive disorders was found at follow-up 24-36 months years after surgery, but not at follow-up 6 to 12 months after surgery [30]. The lack of association between weight loss and improvement in quality of life and the finding of no differences in weight loss between the three groups of patients are probably explained by the short length of follow-up in our study. In line with earlier research we can speculate that such associations between weight loss and quality of life and between weight loss and post-operative psychiatric co-morbidity will be obvious in studies with longer than one year follow-up after surgery.

Level of postoperative HRQOL in patients without postoperative psychiatric disorders is comparable to or better than the population norm one year after surgery. These results are somewhat better than expected. It has been suggested that this discrepancy could be explained by short term euphoria due to a long term disability having been relieved. It can also been explained by the fact that the population norm is based on the general population, which includes patients with and without psychiatric disorders. Our group of patients without postoperative psychiatric disorders is probably healthier with regard to psychiatric illness than the general population.

Despite the fact that comorbid psychiatric disorders are strongly associated with impaired HRQOL in patients with severe obesity [10], having comorbid psychiatric disorders does not usually strengthen the indication for bariatric surgery. It has been argued that preoperative comorbid depression should lower the threshold for surgical treatment [49]. This is based on findings in several studies that weight loss in patients with severe obesity is associated with a decrease in mean level of depressive symptoms and a lower prevalence of clinically significant depression [50,51]. The findings in our study indicate that patients with psychiatric disorder that resolve after surgery had large improvements in quality of life, they achieve a HRQOL comparable to the population norm, and in accordance with this, they might be given priority for bariatric surgery. However, further research is needed to identify maintaining factors for psychiatric disorders in patients undergoing bariatric surgery, in order to develop effective individual psychosocial intervention strategies to improve outcome.

### Strengths and limitations

One strength of the present study is that comorbid psychiatric diagnoses are based on structured diagnostic interviews both prior to and after surgery. In addition, we used a well-validated measure of HRQOL. Norwegian normative data were available for SF-36, which made it possible to compare this study population with the general population.

Some limitations of the study should be considered. One year follow-up may not be a long enough time to capture long-term maintenance of outcome. Changes in HRQOL are reported to follow phases of weight loss, weight regain and weight stability after bariatric surgery. Thirty-one percent of the patients did not complete assessment at follow-up one year after surgery. It was difficult to motivate patients for follow-up, despite repeated mailings and phone calls. High rates of attrition are relatively common in longitudinal obesity studies [30,52]. We found no differences in sociodemographic data, BMI, prevalence of comorbid somatic or psychiatric disorders, or in preoperative scores on SF-36 subscales between completers and non-completers of the follow-up one year after surgery. Thus, it seems reasonable to assume that our findings are representative of patients who seek bariatric surgery. The observed longitudinal change in quality of life may reflect a regression-to-mean phenomenon for patients without psychiatric disorders and patients with psychiatric disorders that resolve after surgery. For patients with the initially lowest scores on the SF-36 subscales (patients with psychiatric disorders that persist after surgery) regression-to-mean did not appear to be a factor. However, the main objective of this study was to compare postoperative quality of life with the population norm in patients without psychiatric disorders, patients with psychiatric disorders that resolve after surgery, and patients with psychiatric disorders that persist after surgery. Finally, we acknowledge that a disease-specific HRQOL measure could have complemented our results.

## Conclusion

In the present study we found a significant improvement in HRQOL from preoperative assessment to follow-up one year after bariatric surgery. Patients without postoperative psychiatric disorders achieved a HRQOL comparable to the general population one year after bariatric surgery. However, in patients with postoperative psychiatric disorders, only a trend toward improvement could be detected in several of the subscales that measured HRQOL, and these patients still had impaired HRQOL compared to the population norm one year after surgery. Our results support surveillance of patients with psychiatric disorders that persist after surgery for suboptimal improvements in quality of life after bariatric surgery.

## Competing interests

The authors declare that they have no competing interests. The authors alone are responsible for the content and writing of the paper.

## Authors' contributions

HØL participated in the design of the study, collected the data, performed the statistical analysis, and drafted the manuscript. EB contributed to the process of designing the study, provided advice and follow-up during the collection of data, and has given supervision regarding data analysis and writing of the manuscript. OH participated in the analysis and interpretation of data and helped to draft the manuscript. BS and TT contributed to the process of designing the study and helped to draft the manuscript. All authors read and approved the final version of the manuscript.
